# Cortical Statistical Correlation Tomography of EEG Resting State Networks

**DOI:** 10.3389/fnins.2018.00365

**Published:** 2018-05-30

**Authors:** Chuang Li, Han Yuan, Guofa Shou, Yoon-Hee Cha, Sridhar Sunderam, Walter Besio, Lei Ding

**Affiliations:** ^1^School of Electrical and Computer Engineering, University of Oklahoma, Norman, OK, United States; ^2^Stephenson School of Biomedical Engineering, University of Oklahoma, Norman, OK, United States; ^3^Institute for Biomedical Engineering, Science and Technology, University of Oklahoma, Norman, OK, United States; ^4^Laureate Institute for Brain Research, Tulsa, OK, United States; ^5^Department of Biomedical Engineering, University of Kentucky, Lexington, KY, United States; ^6^Department of Electrical, Computer, and Biomedical Engineering, University of Rhode Island, Kingston, RI, United States

**Keywords:** EEG, fMRI, resting state network, inverse source imaging, ICA, statistical correlation

## Abstract

Resting state networks (RSNs) have been found in human brains during awake resting states. RSNs are composed of spatially distributed regions in which spontaneous activity fluctuations are temporally and dynamically correlated. A new computational framework for reconstructing RSNs with human EEG data has been developed in the present study. The proposed framework utilizes independent component analysis (ICA) on short-time Fourier transformed inverse source maps imaged from EEG data and statistical correlation analysis to generate cortical tomography of electrophysiological RSNs. The proposed framework was evaluated on three sets of resting-state EEG data obtained in the comparison of two conditions: (1) healthy controls with eyes closed and eyes open; (2) healthy controls and individuals with a balance disorder; (3) individuals with a balance disorder before and after receiving repetitive transcranial magnetic stimulation (rTMS) treatment. In these analyses, the same group of five RSNs with similar spatial and spectral patterns were successfully reconstructed by the proposed framework from each individual EEG dataset. These EEG RSN tomographic maps showed significant similarity with RSN templates derived from functional magnetic resonance imaging (fMRI). Furthermore, significant spatial and spectral differences of RSNs among compared conditions were observed in tomographic maps as well as their spectra, which were consistent with findings reported in the literature. Beyond the success of reconstructing EEG RSNs spatially on the cortical surface as in fMRI studies, this novel approach defines RSNs further with spectra, providing a new dimension in understanding and probing basic neural mechanisms of RSNs. The findings in patients' data further demonstrate its potential in identifying biomarkers for the diagnosis and treatment evaluation of neuropsychiatric disorders.

## Introduction

Over the past decade, research on the human brain has been increasingly drawn toward investigation of networked brain activity among different brain regions during resting states, termed as the resting state networks (RSNs) (Biswal et al., 1995; Fox and Raichle, 2007). In contrast to task-related brain activities (Jensen and Tesche, 2002; Dosenbach et al., 2007), RSNs reflect the intrinsic functional organization and rhythm of the human brain when it is not engaged in any task or disturbed by external stimuli (Di et al., 2013; Cole et al., 2014, 2016). Technically, RSNs are represented by correlated spontaneous fluctuations of signals generated from distinct brain areas (Biswal et al., 1995; Lowe et al., 1998; Raichle et al., 2001). To date, RSNs have been widely studied using functional magnetic resonance imaging (fMRI) (Biswal et al., 1995; Di Martino et al., 2008; Van Den Heuvel and Pol, 2010), which measures blood oxygenation level-dependent (BOLD) signals. Resting state fMRI studies have identified various RSNs associated with different brain functions (De Luca et al., 2006), demonstrated their consistency in healthy subjects (Beckmann et al., 2005; Damoiseaux et al., 2006), alterations in neuropsychiatric disorders (Rombouts et al., 2005; Greicius et al., 2007; Sorg et al., 2007; Agosta et al., 2012), and changes with cognitive tasks (Greicius et al., 2003; Buckner et al., 2008).

BOLD signals, however, are not direct measurements of neuronal electrical activity (Attwell and Iadecola, 2002), and their low temporal resolution cannot sufficiently characterize temporal or spectral information of fast neural oscillations (Logothetis, 2008). Therefore, BOLD fMRI is limited in revealing the underlying electrophysiological basis of RSNs, which are further impacted by the fact that the hemodynamic process is affected by respiration and circulation (Logothetis et al., 2001; Cole et al., 2010). On the contrary, electroencephalography (EEG) and magnetoencephalography (MEG) directly measure neuronal activity. Their excellent temporal resolution allows examination of neuronal changes on the millisecond timescale (Hämäläinen et al., 1993; Laufs et al., 2003b). Various computational techniques have been recently developed to detect RSNs from EEG or MEG data alone, resulting in spatial patterns with significant similarity to RSNs derived independently from fMRI data (Brookes et al., 2011; Ramkumar et al., 2012; Yuan et al., 2016; Liu et al., 2017).

Among various methods for probing resting-state brain signals, independent component analysis (ICA) has been widely used to identify RSNs from both EEG/MEG and fMRI data. To achieve better computational accuracy of ICA, the dimension with the larger size is usually chosen as the sample domain (Hyvärinen et al., 2004). In particular, spatial ICA is often used on fMRI data (Beckmann et al., 2009; Calhoun et al., 2009) because the spatial dimension of fMRI data is relatively larger than its temporal dimension. Temporal ICA is widely performed on EEG/MEG data (Brookes et al., 2011; Yuan et al., 2016) because of their relatively large temporal dimensions. In order to spatially define EEG/MEG RSNs on the cortical surface, i.e., the same domain of fMRI RSNs, ICA has been used in combination with EEG/MEG inverse source imaging (ISI) techniques (Mosher et al., 1999; Pascual-Marqui, 1999; Grech et al., 2008; Yuan et al., 2016). In ISI, resting state EEG/MEG data are subject to inverse reconstruction of underlying sources on the cortical surface. Resulted cortical source data are analyzed by ICA to reconstruct a network-level organization of activities. It is noted that these techniques make it feasible to directly compare fMRI RSNs and EEG/MEG RSNs in a common spatial domain, providing new insights to the electrophysiological basis and hemodynamic aspects of RSNs, especially when fMRI and EEG data can be simultaneously recorded (Goldman et al., 2002; Gonçalves et al., 2006; Yuan et al., 2016).

Despite these new advancements, methods to derive EEG/MEG RSNs are still limited in many ways. First, the mathematical principle of ICA favors the detection of non-Gaussian distributed components, making it a very successful tool in finding artifacts rather than components related to brain activity (Hyvärinen and Oja, 2000; Vigário et al., 2000), which is certainly not optimized for separating networks of organized neural activity. Second, spatial patterns defined with linear mixing weights of ICA for independent components (ICs) have been shown to be less optimal than those obtained through an additional correlation analysis between source time series and IC time series (Brookes et al., 2011; Yuan et al., 2016). Third, ICA mixing weights are lack of statistical meanings to be systematically assessed in defining the spatial coverage of an RSN. Fourth, most brain activity is rhythmic in nature and spectral characteristics of individual RSNs have been assumed *a priori* using pre-selected band-pass filters (Mantini et al., 2007; Brookes et al., 2011). However, such a strategy is not always optimal and could introduce bias, especially when an RSN has a broadband spectral pattern and various power spectra across individuals. Finally, in group analysis with multiple individuals, inter-individual variance exists in resting state analysis (Gonçalves et al., 2006), but it has not been addressed when data from all individuals are simply concatenated for analysis by the ICA routine.

In the present study, a new framework termed time-frequency ICA-based statistical correlation tomography (TFICA-SCT) is proposed to identify RSNs from EEG data by combining ISI, a unique time-frequency ICA method, and statistical correlation analysis. ISI was performed on complex-valued EEG data after a short-time Fourier transform (STFT) to reconstruct cortical source maps. Instead of conventional temporal ICA (TICA), TFICA was implemented by applying an ICA algorithm on the time-frequency representation (TFR) of cortical source data from ISI. The correlation analysis between IC time series and source time series from ISI was further used to obtain optimal spatial patterns of RSNs. A series of steps was further developed and conducted to reconstruct genuine tomography of RSNs, which included processes to address inter-individual variance, conversion to a statistical metric using the Fisher's z-transform, correction of false cross-correlation from autocorrelation, and thresholding by a cluster-based statistical approach.

The present study examined the performance of TFICA-SCT using three sets of experimental data from both healthy and symptomatic participants. Derived tomographic maps were compared with templates from resting state fMRI to evaluate their consistency with RSN definitions from an independent neuroimaging modality. The robustness of the method in identifying RSNs in both spatial and spectral patterns was evaluated by comparing results obtained from three datasets. In each data set, there were two conditions (i.e., eyes-open vs. eyes-closed; healthy individuals vs. patients; and before vs. after treatment) and their comparisons were used to examine the resolution and capability of the proposed framework in identifying condition-specific differences.

## Materials and methods

### Cortical statistical correlation tomography of RSNs

#### Forward models and lead field computation

**Figure 1:**
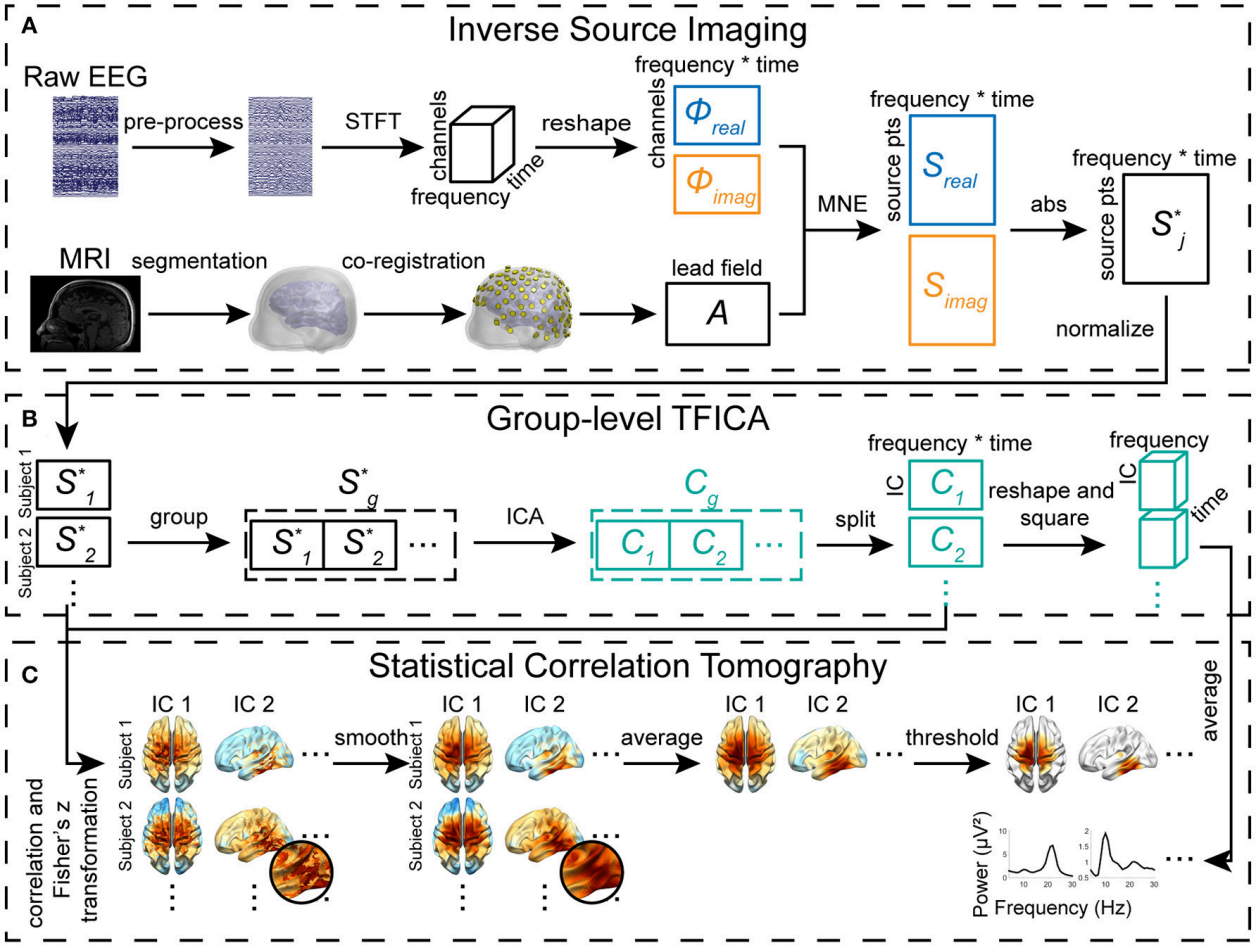
Diagram of TFICA-SCT. **(A)** Inverse source imaging, including EEG preprocessing, STFT, volume conduction modeling, BEM forward calculation of lead field, and MNE inverse solution. **(B)** Group-level TFICA on the cortical source domain. **(C)** Statistical correlation tomography derived by correlational analysis of time courses between ICs and sources.

The cortical current density (CCD) source model (Dale and Sereno, 1993) was used in the present study, in which the source space was represented numerically by continuously distributed triangular elements over the cortical surface (Figure 1). The anatomical cortical model of each participant was obtained by segmenting the white matter/gray matter interface from the participant's head magnetic resonance imaging (MRI) using FreeSurfer (Fischl, 2012). The cortical surface was triangulated into a high-resolution mesh of 40,960 triangles (Figure 1). Each dipole source was placed at the center of a triangle on the cortical mesh and its orientation was perpendicular to the corresponding triangle. Boundary element (BE) volume conductor models were used to represent the realistic geometrical shape of the human head and major conductivity profile (e.g., the scalp, skull, and brain) for the forward problem calculation. The BE models have 10,240 triangles in each of three surface meshes, which were obtained by segmenting the surfaces of the scalp, skull, and brain from structural MRI and were assigned different conductivities (0.33/Ωm, 0.0165/Ωm, and 0.33/Ωm, respectively) (Lai et al., 2005). Co-registration of BE models and electrodes was implemented by aligning three landmarks, i.e., nasion, left and right pre-auricular points.

The forward model can be expressed in the following equation:

(1) Φ=A·S+N

where **Φ** is a matrix of recorded EEG signals at electrodes each as a function of time, ***S*** is the unknown matrix of amplitudes of dipole sources at triangles on CCD each as a function of time, and ***A*** is the lead field matrix linking dipole sources with EEG recordings, which was calculated using the boundary element method (BEM) (Hämäläinen and Sarvas, 1989) based on the defined source model, volume conductor model, and co-registered EEG electrode locations. *N* is noise.

#### Inverse source imaging

ISI was performed in the complex domain of recorded EEG data to prepare inverse source data for the analysis by TFICA. The STFT was applied on non-overlapping 1 s epoch EEG data, resulting in a 3D TFR of EEG data, i.e., channel, frequency, and time (or epoch). Twenty-seven frequency bins from 4 to 30 Hz were kept with a resolution of 1 Hz. The 3D TFR was then reshaped into a 2D matrix by concatenating the dimensions for frequency and epoch.

Since STFT is a linear transformation, it does not change the relationship between dipole sources and EEG recordings in Equation (1), while it leads to two values, i.e., real and imaginary parts, for each measurement at an electrode. Therefore, Equation (1) was re-arranged as:

(2) $$\left[\begin{array}{c} \Phi_{real}\\ \Phi_{imag} \end{array}\right]=\left[\begin{array}{cc} A & 0\\ 0 & A \end{array}\right]\cdot \left[\begin{array}{c} S_{real}\\ S_{imag} \end{array}\right]+\left[\begin{array}{c} N_{real}\\ N_{imag} \end{array}\right]$$

The lead field matrix is same for both real and imaginary parts. **Φ** and ***S*** were redefined as $\Phi =\left[\begin{array}{c} \Phi_{real}\\ \Phi_{imag} \end{array}\right]$ and $S=\left[\begin{array}{c} S_{real}\\ S_{imag} \end{array}\right]$. Since the number of discrete sources was larger than the number of measurements (i.e., the number of electrodes), regularized solutions were needed to produce stable results (Michel et al., 2004). In the present study, a minimum-norm estimate (MNE) (Hämäläinen and Ilmoniemi, 1984; Dale and Sereno, 1993) of dipole amplitudes in the sense of L2-norm at each time-frequency point was obtained by:

(3) $$min\left(||\Phi -A\cdot S|{|}^{2}+\lambda \cdot ||S|{|}^{2}\right)$$

where ||·|| indicates the L2-norm and λ is the regularization parameter. Minimization in Equation (3) over Φ yields the MNE estimate of *S*, i.e., *S'*, in the following form:

(4) $$S^{\prime }=T\cdot \Phi$$

And ***T*** is a linear inverse operator to measured signals expressed by:

(5) $$T={A_{H}}^{T}\cdot {\left(A_{H}\cdot {A_{H}}^{T}+\lambda \cdot H\right)}^{-1}$$

where ***A***_***H***_ **=** ***H*** **·** ***A*** and ***H*** **=** ***I*****−11**^***T***^**/1**^***T***^**1** is the average reference operator (Pascual-Marqui, 1999), *I* is the identity matrix, and ***1*** is a vector of all ones. The selection of the regularization parameter λ was achieved using the generalized cross validation (GCV) method (Golub et al., 1979; Wahba, 1990). It was noted that the use of the matrix *H* was to address the issue with the use of the reference, which makes source estimations reference free similar to the reference electrode standardization technique (Yao, 2001, 2017). The reason of calculating ISI in the frequency domain had three-fold. First, it met the need of time-frequency data in the subsequent TFICA method (see below); second, it could reduce computational loads; Third, the ISI in the frequency domain has indicated with superior performance on rhythmic EEG signals (Yuan et al., 2011).

#### Group-level TFICA

After obtaining source data from all subjects, ICA analysis was carried out at the group level. First, amplitudes of source data at individual time points were obtained as absolute values of complex time courses. Then, data from individuals were normalized using a z-transform to reduce inter-individual variations, yielding normalized source amplitude data, i.e., S^*^. Afterwards, individual source data from a group of participants were concatenated in the tempo-spectral domain, leading to a group-level 2D source matrix $S_{g}^{*}$ [channels × (participants, epochs, frequency bins)], in which the subscript *g* indicates the group-level matrix. The Infomax ICA (Lee et al., 1999) from EEGLAB (Delorme and Makeig, 2004) was applied on $S_{g}^{*}$ to obtain group-level independent components (*C*_*g*_), each representing a cortical map of brain activities involving multiple regions (nodes) mutually dependent and forming a network, as:

(6) $$C_{g}=W^{-1}\cdot S_{g}^{*}$$

where ***W*** is the mixing matrix. The number of underlying sources had been suggested to be around 30 (Smith et al., 2009; Abou-Elseoud et al., 2010; Ramkumar et al., 2014). A relatively larger number of ICs (i.e., 40) was pre-selected in the present study. Each column in ***C***_***g***_ represents the linear mixing weights from all dipole source points (i.e., 40,960), and each row represents concatenated time-frequency courses from all participants for the corresponding IC.

#### Statistical correlation tomography

To define spatial patterns of each IC on the cortical surface, columns of *C*_*g*_ were first segmented to form the *C*_*i*_ matrix for each participant, as in Equation (7). Correlation coefficients (CCs) between the time-frequency course of each IC (i.e., each row of *C*_*i*_) and the time-frequency course of each source on the CCD model (i.e., each row of $S_{i}^{*}$) were calculated for all individual participants in the group, as in Equation (8). CC values were converted into z-values using the Fisher's z-transformation (Fisher, 1915) according to Equation (9).

(7) $$\begin{array}{l} C_{g}=\left[C_{1},C_{2},\ldots ,C_{i},\ldots ,C_{N}\right],i=1,\ldots N\;and\\ \;N\;=\;number\;of\;participants \end{array}$$

(8) $$\begin{array}{l} CC_{i}\left(j,k\right)=corr\left(C_{i}\left(j\right),S_{i}^{*}\left(k\right)\right),j=1,\ldots ,40;\\ \;k=1,\ldots ,40,960 \end{array}$$

(9) $$\begin{array}{l} z_{i}(j,k)=\frac{1}{2}ln\left(\frac{1+CC_{i}(j,k)}{1-CC_{i}(j,k)}\right),j=1,\ldots ,40;\\ \;k=1,\ldots ,40,960 \end{array}$$

All correlation maps were then smoothed by an iterative smoothing algorithm, i.e., the heat kernel smoothing with full width at half maximum of 8 mm (Chung et al., 2005), which has been widely used in fMRI (Hagler et al., 2006; Chung et al., 2010). It is known that cross correlations calculated in Equation (8) are impacted by autocorrelations in either ***C***_***i***_***(j)*** or $S_{i}^{*}$(k) signals (Friston et al., 1994). Therefore, autocorrelations of ***C***_***i***_***(j)*** and $S_{i}^{*}$(k) were computed to correct z-values in Equation (9) via adjusting the degree of freedom (DOF) according to the Bartlett's theory (Bartlett, 1935):

(10) $$\begin{array}{l} {N_{i}}^{\prime }(j,k)=N_{i}(j,k)\cdot \frac{1-\rho_{C}(j,k)\cdot \rho_{S}(j,k)}{1+\rho_{C}(j,k)\cdot \rho_{S}(j,k)},\;i=1,\ldots P\;and\\ \;P=number\;of\;participants \end{array}$$

where the **ρ**_***C***_ and **ρ**_***S***_ are the autocorrelation coefficients of ***C***_***i***_ and $S_{i}^{*}$, respectively. Thus, the square root of the theoretical variance of z_*i*_(*j*,*k*) is $1/\sqrt{N_{i^{\prime }}(j,k)-3}$. By dividing it, z-values were converted into z-scores (that is, zero mean, unit variance, Gaussian distributions under the null hypothesis of no correlation) (Vincent et al., 2007) for each IC, the group-level z-score maps were calculated using an approach that had been suggested more effective on relatively small samples (Silver and Dunlap, 1987; Alexander, 1990).

(11) $$\begin{array}{l} \overset{¯}{z}(j,k)=\frac{\Sigma \left(N_{i}^{\prime }(j,k)-3\;\right)\cdot z_{i}}{\Sigma \;\left(N_{i}^{\prime }(j,k)-3\right)},\;i=1,\ldots N\;and\\ \;N=number\;of\;participants \end{array}$$

#### Cluster-based statistical thresholding

To quantitatively define brain regions that belong to an RSN (i.e., an IC), statistical correlation coefficient (SCC) maps obtained after Equation (11) need to be thresholded. This was done by applying a *t*-test against zero on z-scores from all participants in a group at each source on the CCD model. The threshold was set at *p* < 0.01 to create a binary mask with 1 for locations of significant correlations. To address the multiple comparisons problem, a cluster-based correction method was employed (Hagler et al., 2006). Specifically, in a Monte Carlo simulation, random brain signals were generated on the CCD model and then the steps starting from Equations (8) to (10) were followed to create pseudo-SCC maps thresholded at *p* < 0.01 as in real data. The process was repeated 1,000 times to create a histogram for the size of clusters (connected areas on the CCD mesh) under the null hypothesis. From the histogram, the threshold of cluster size for real data was identified at *p* < 0.01, and used to remove small clusters to reduce false positives (i.e., removing clusters on binary masks smaller than the threshold). The same procedure was performed on all ICs to create SCT for each RSN.

### Experimental protocols

To evaluate the performance of the proposed TFICA-SCT, systematic evaluations were conducted in three sets of experimental datasets acquired from both healthy individuals and individuals with a balance disorder called mal de debarquement syndrome (MdDS), which is described below. The datasets included (1) resting EEG in seven healthy individuals in eyes-closed (EC) and eyes-open (EO) conditions, termed as the EC/EO dataset; (2) resting EEG in seven healthy controls (HC) and seven individuals with MdDS termed as the HC/MdDS dataset; and (3) resting EEG in seven MdDS individuals before and after receiving treatment of repetitive transcranial magnetic stimulation (rTMS), termed as the Pre-/Post-rTMS dataset. The study protocol and acquisition settings of these experimental data have been detailed in our prior work (Ding et al., 2014; Yuan et al., 2017) and they are briefly described below.

#### Participants

Written informed consent was obtained from all participants before the study. All study procedures were approved by the Western Institutional Review Board (www.wirb.com). To examine the proposed tomography, the present study included data from seven healthy controls (all females; age: 51.1 ± 8.0 years) and seven patients (all females; age: 53.1 ± 12.1 years) with MdDS (Cha, 2009; Ding et al., 2014). The recruitment of participants of MdDS did not exclude male participants, but the prevalence of females is much higher than males in MdDS (Cha, 2009). Thus, the matched healthy control population only included female participants as well.

MdDS is caused by exposure to oscillating environments such as a flight or a cruise, leading to persistent sensation of rocking dizziness (Cha, 2009, 2015; Cha et al., 2012, 2013). It is the unnatural persistence of the natural phenomenon of motion entrainment. rTMS treatment had been demonstrated with therapeutic effects in MdDS (Cha et al., 2013, 2016a,b; Ding et al., 2014). All MdDS patients in the present study received five consecutive days with one session on each day. The rTMS target in all patients was the dorsolateral prefrontal cortex (DLPFC), which was located by the Localite TMS Navigator (Localite GmBH, Germany) frameless stereotaxy system. The Magventure MagPro X100 stimulator (MagVenture A/S, Farum, Denmark) was used to generate magnetic stimulation pulses including 1 Hz right DLPFC stimulation of 1,200 pulses followed by 10 Hz left DLPFC of 2,000 pulses. The treatment effects were evaluated using a clinical visual analog scale (VAS) (Cha et al., 2013; Shou et al., 2014; Yuan et al., 2017).

#### EEG recording

A BrainAmp amplifier (Brain Products GmbH, Munich, Germany) was used to record resting-state EEG signals from 126 channels at a sampling frequency of 1,000 Hz. The ground electrode was placed at AFz and FZ was chosen as the recording reference channel. Participants were recorded while lying quietly with eyes closed. Participants in the HC group performed two 5-min sessions of simultaneous fMRI-EEG recordings with their eyes open and closed, respectively. It should be noted that only EEG data were analyzed in the present study. Participants with MdDS underwent two 5-min sessions of EEG, one before the first TMS session (Pre-TMS) on the first day and the other 4–5 h after the last TMS on the 5th day (Post-TMS). Since the effect of rTMS on MdDS patients has been investigated in our previous study (Ding et al., 2014), the inclusion of EEG data from MdDS patients before and after rTMS in the present study served as a contrast to examine the sensitivity of the proposed approach.

#### MRI

Structural magnetic resonance images of participants' heads were obtained using a General Electric Discovery MR750 whole-body 3-Tesla MRI scanner (GE Healthcare, Milwaukee, Wisconsin) through a T1-weighted magnetization-prepared rapid gradient echo (3D MPRAGE) sequence. The parameters for imaging were: FOV = 240 mm, axial slices per slab = 190, slice thickness = 0.9 mm, image matrix = 256 × 256, TR/TE = 5/2.012 ms, acceleration factor R = 2, flip angle = 8°, inversion time TI = 725 ms, sampling band-width = 31.2 kHz.

### Data analysis protocols

#### Preprocessing of EEG

EEG data from each participant was preprocessed with the following pipeline. First, a band-pass filter of 0.5–100 Hz and a notch filter of 60 Hz was used on EEG data. Second, bad channels were detected using the FASTER toolbox (Nolan et al., 2010) with visual inspection as a supplemental check, followed by interpolation from neighboring channels using EEGLAB (Delorme and Makeig, 2004). Third, EEG data were divided into non-overlapping 1 s epochs (same as those being analyzed using STFT in section Inverse Source Imaging). Bad epochs were rejected using the FASTER toolbox. During detections of bad channels and bad epochs, a threshold at z-score > 3 was selected. Fourth, the Infomax ICA (Lee et al., 1999) was utilized to decompose time-domain EEG data concatenated from all epochs into 64 ICs. Basing on visual inspection of spatial and spectral features of all ICs, ICs linked to ocular, cardiac and muscular activities were removed. Finally, denoised EEG data were re-referenced to a common average and down-sampled to 250 Hz. For EEG data collected with fMRI, the same framework of preprocessing was employed as in our previous studies (Yuan et al., 2012, 2016), and an additional notch filter of 26 Hz was used to reject vibration noise from the MRI system (Ritter and Villringer, 2006; Mayeli et al., 2016).

#### Analysis by TFICA-SCT

To directly compare tomography from different conditions, the TFICA step in the proposed framework was performed on combined EEG data from the two compared conditions for each group: eyes open vs. eyes closed in HC, HC vs. MdDS, and pre-TMS vs. post-TMS in MdDS. Three SCT analyses were performed on grouped data of participants in both conditions included, and averaged SCTs of ICs were obtained.

#### Spectral powers of ICs

Spectral powers of all ICs were calculated in each participant by reshaping the 2D matrix *Cg* into a 3D matrix (channel × frequency × epoch), squaring all values, and averaging over epochs. These steps resulted in 40 power spectra for 40 ICs at 27 frequency bins ranging from 4 to 30 Hz.

#### Selection of ICs

Brain activity-related ICs were selected from all 40 ICs based on their spatial-spectral features. Specifically, corresponding SCTs of ICs were compared with RSNs defined from resting-state fMRI data (Yeo et al., 2011). Based on the anatomic locations of fMRI RSNs, EEG RSNs were identified mainly for visual, auditory, somatomotor, frontoparietal, and default mode networks. In the spectral domain, theta, alpha, and beta peaks were analyzed with reference to spectral features of RSNs (Mantini et al., 2007) and/or the general 1/f spectra (Freeman et al., 2000; Robinson et al., 2001) were treated as reasonable patterns, while spectral patterns of sharp and narrow peaks and over-oscillations were used in rejecting ICs as artifacts.

### Evaluation and validation protocols

We conducted a series of validations steps on the performance of the TFICA-SCT method. First, RSNs defined through TFICA-SCT were compared with RSN templates derived from fMRI data (Yeo et al., 2011). Second, the SCTs from the three analyses were compared quantitatively to assess the spatial consistency of the obtained results. Third, SCTs from different conditions (i.e., EC vs. EO in the healthy, HC vs. MdDS group, Pre-rTMS vs. Post-rTMS in MdDS) were compared statistically in terms of both spatial and spectral patterns. Details are described below.

#### Comparisons with fMRI RSN templates

To evaluate spatial patterns of obtained EEG RSNs, cortical maps from TFICA-SCT were compared with the five RSN templates from fMRI (Yeo et al., 2011) including visual, auditory, somatomotor, frontoparietal, and default mode networks. For each binary fMRI template and each SCT, the comparison was quantified by a template-matching method (Greicius et al., 2007) with normalization as follows:

(12) $$TD(t,c)=\frac{Z_{in}(t,c)-Z_{out}(t,c)}{Z_{in}(t,c)+Z_{out}(t,c)}$$

where ***TD*** is the template-matching degree; ***t*** is the index for the binary template and *c* is the index of the thresholded SCT. ***Z***_***in***_ is the averaged z-score from source points on SCT inside the fMRI template and ***Z***_***out***_ is the averaged z-score from source points outside. TDs were calculated for all possible pairs between selected RSNs and the five templates. The significance of TD was evaluated by a Monte Carlo simulation. Specifically, for a template-SCT pair, the z-scores on each source point were randomly shuffled 500,000 times and the corresponding TDs were computed, generating a histogram of TDs. Based on the histogram, the *p* value was obtained for each pair and the significance level was 0.01, with Bonferroni correction. Positive TDs confirmed the spatial similarity between templates and SCTs. To investigate possible confusion in matching templates and SCTs, positive TDs within- and between-class were compared using *t*-tests across five networks and three analyses.

#### Evaluation of spatial consistency in multiple datasets

To evaluate spatial pattern consistency of EEG RSNs obtained from multiple datasets, the spatial patterns of SCTs obtained from the three analyses (i.e., EC vs. EO in the healthy, HC vs. MdDS, Pre-rTMS vs. Post-rTMS in MdDS) were quantitatively compared in pairs using the metric of TD as follows:

(13) $$\bar{TD}\left(i,j\right)=\frac{TD(i,j)+TD(j,i)}{2}$$

where in ***TD(i,j)*** the threholded SCT ***i*** from one analysis was binarized and used as the template to calculate TD against the thresholded SCT ***j*** from another analysis. The same analysis was repeated after two SCTs were shifted where the thresholded SCT ***j*** was binarized. The TD between the SCTs ***i*** and ***j*** was taken as the average of two analyses, resulting in a TD map for all possible pairs. Since the SCTs were separated into five classes of RSNs, there were twenty-five large tiles in a TD map, each containing TD data for RSN-RSN pairs in one class. In the matrix representation of TD maps (see Figure 6 for an example), the diagonal tiles represented within-class TDs while off-diagonal tiles contained cross-class TDs. As in the above section, positive TDs within- and between-class were compared using a *t*-test.

#### Difference in statistical correlation tomography

To probe differences between two conditions in each dataset, the SCTs for data from two conditions were re-calculated separately (Equations 8–12) based on the group-level TFICA results and then statistically compared. In each dataset for each RSN (defined by an IC), regions with significant differences were detected by applying a two-tailed *t*-test at each source point between two SCTs within binary masks defined in the section Cluster-Based Statistical Thresholding. The resulted clusters of difference on the CCD model were subject to the same cluster-based correction method to control false positives. For visualization, identified clusters of difference on the CCD model from two compared conditions were displayed as the difference of SCC values between two SCTs.

#### Power spectra difference

Power spectra of individual ICs were used as the second metric to evaluate the difference between two compared conditions. At each individual subject and for each selected IC, a two-tailed *t*-test was used to compare specific band power at theta, alpha or beta bands between conditions over all epochs, determining whether there were significant differences between two conditions and the direction of difference (i.e., increase or decrease). The analysis was only performed on two datasets (i.e., EC vs. EO in the healthy controls and pre-rTMS vs. post-rTMS in MdDS) since the third dataset (i.e., HC vs. MdDS) involved different subject groups precluding the direct statistical comparison. Significant increase/decrease was collected from all subjects for each IC at each frequency band and aggregated for each class of RSNs, yielding data for five RSNs and three frequency bands (Table 1). For each RSN class at each band, the number of increase/decrease was tested against the number of total significant changes at the group level using a binomial test (hypothetical probability of increase/decrease = 0.5).

**Table 1:** Summary of spectral power differences of five RSN classes between data from two conditions in one dataset: (A) EC/EO and (B) Pre-/Post-rTMS.

	**↑**	***p*-value**	**↓**	***p*-value**
**EC/EO**				
**THETA**				
V	3	1.00	22	<**0.001**
A	11	0.33	10	0.5
M	0	1.00	11	<**0.001**
F	20	0.06	12	0.89
D	2	0.66	4	0.11
**ALPHA**				
V	3	0.998	16	<**0.001**
A	14	0.154	10	0.729
M	1	0.980	8	<**0.005**
F	29	<**0.001**	7	1.00
D	1	0.688	3	0.06
**BETA**				
V	22	<**0.001**	5	1.00
A	25	<**0.001**	3	1.00
M	5	0.71	8	0.13
F	41	<**0.001**	1	1.000
D	5	<**0.001**	0	0.97
**Pre-/Post-rTMS**				
**THETA**				
V	10	0.32	9	0.5
A	11	<**0.05**	5	0.89
M	9	0.5	10	0.32
F	8	0.68	11	0.18
D	15	0.57	17	0.30
**ALPHA**				
V	11	0.42	11	0.42
A	10	0.17	7	0.69
M	12	0.13	8	0.75
F	12	0.19	9	0.67
D	6	1.00	28	<**0.001**
**BETA**				
V	8	0.86	14	0.07
A	12	0.13	8	0.75
M	11	0.18	8	0.68
F	11	0.18	8	0.68
D	17	0.57	19	0.31

*BOLD: p < 0.05 at least*.

## Results

### Spatial and spectral patterns of RSNs

**Figure 2:**
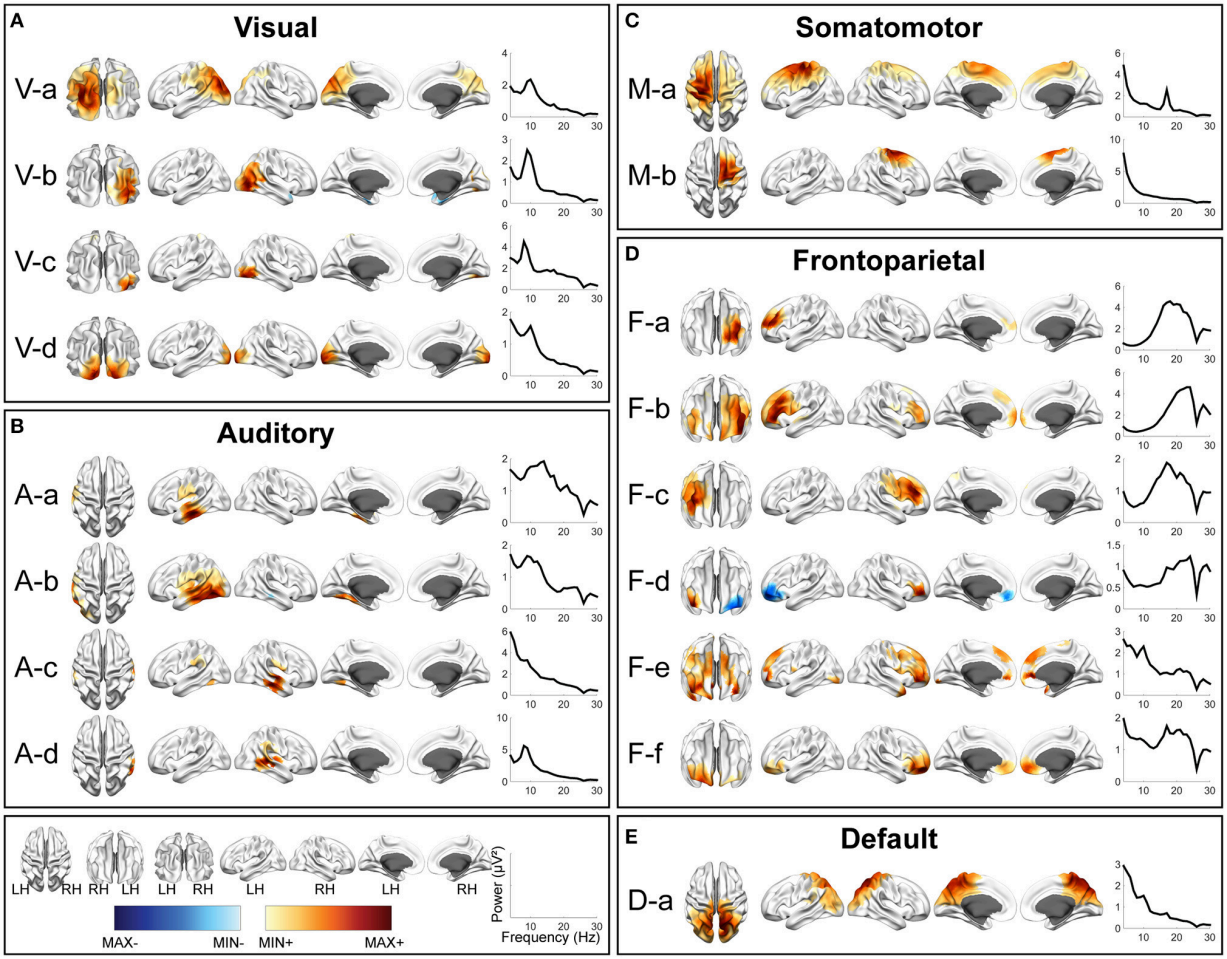
Spatial and spectral patterns of five RSN classes from TFICA-SCT on the EC/EO EEG data. **(A)** Visual, **(B)** auditory, **(C)** somatomotor, **(D)** frontoparietal, and **(E)** default mode networks. Group-level SCT maps were thresholded at *p* < 0.01, cluster-based correction.

**Figure 3:**
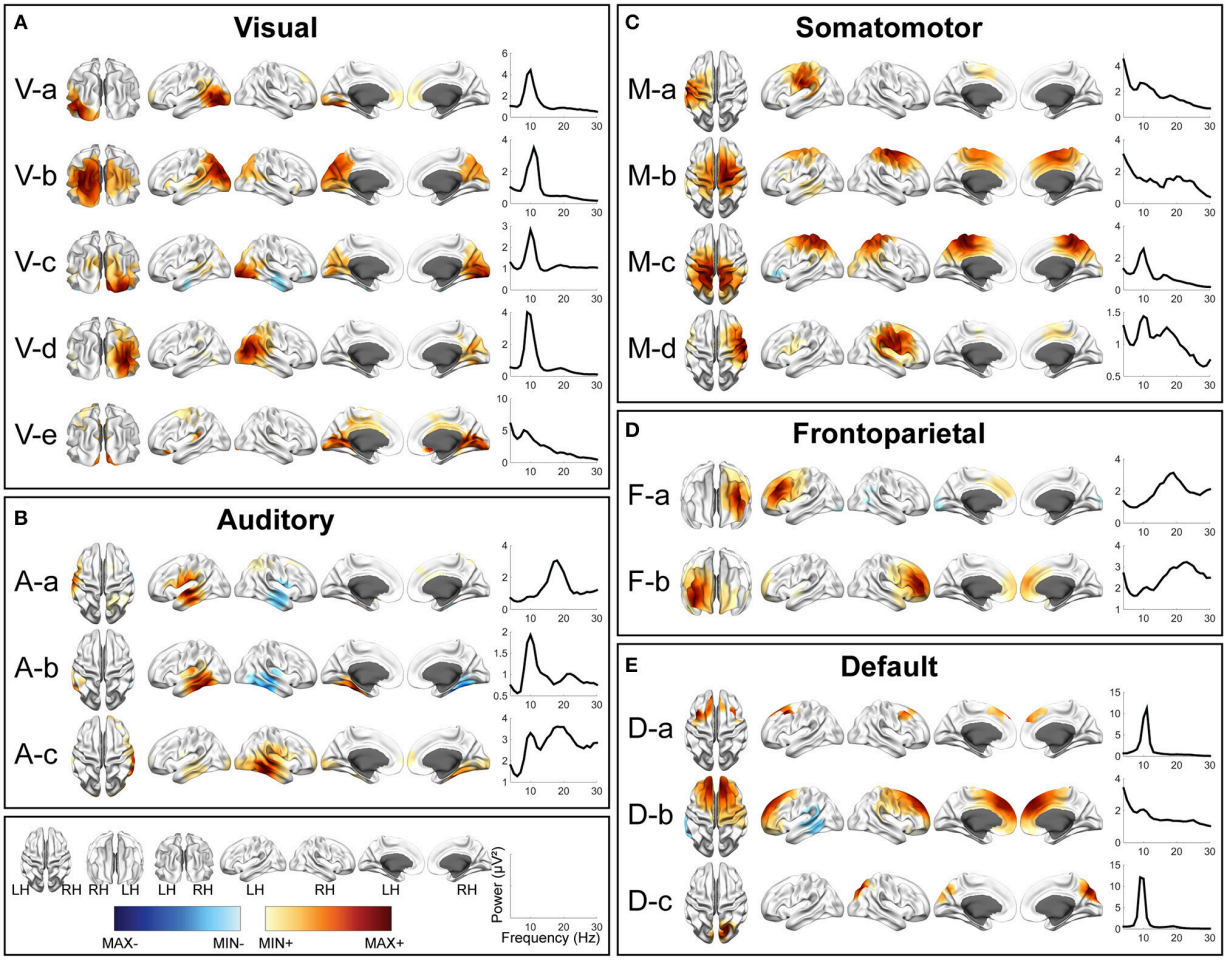
Spatial and spectral patterns of five RSN classes from TFICA-SCT on the HC/MdDS EEG data. **(A)** Visual, **(B)** auditory, **(C)** somatomotor, **(D)** frontoparietal, and **(E)** default mode networks. Group-level SCT maps were thresholded at *p* < 0.01, cluster-based correction.

**Figure 4:**
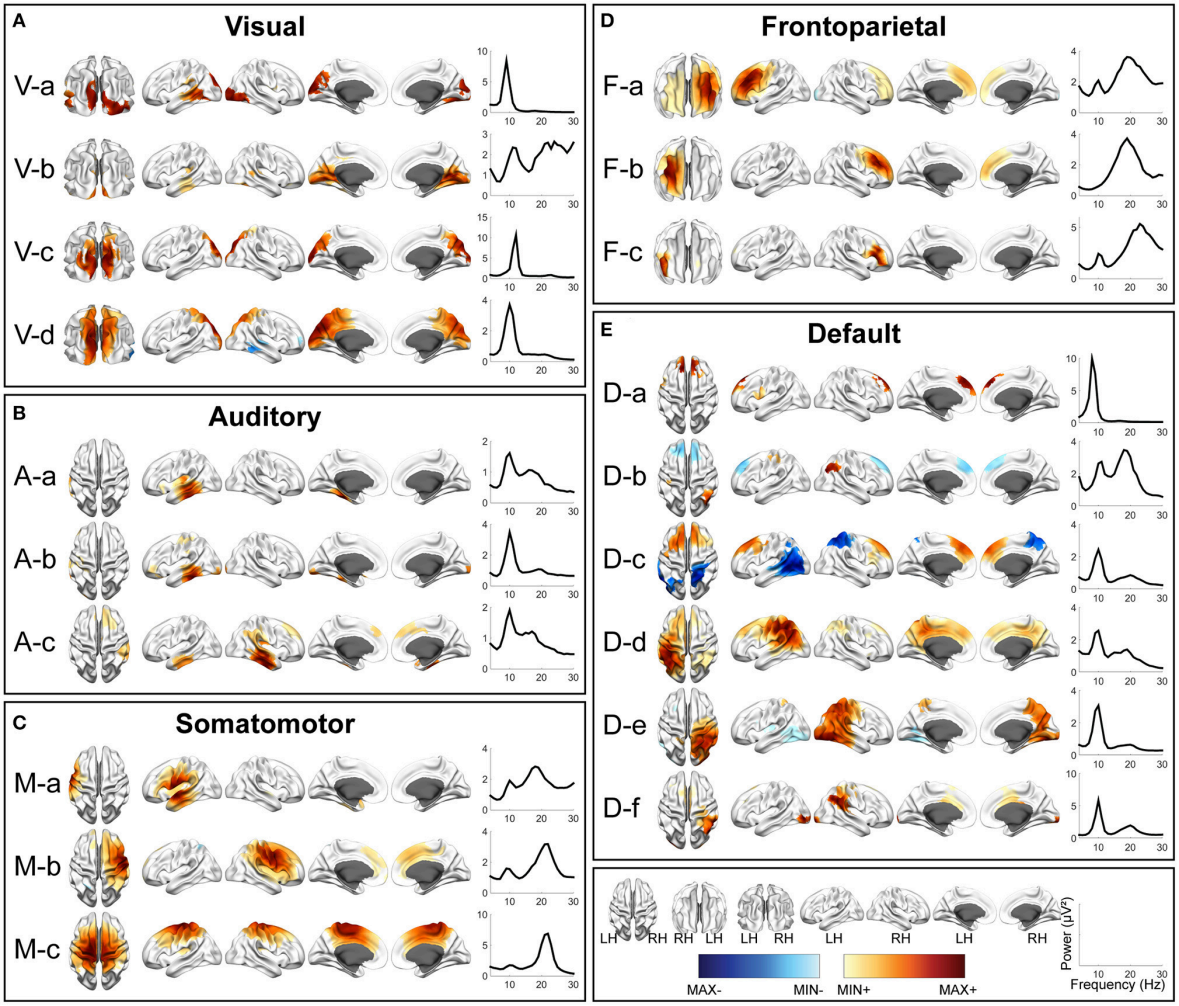
Spatial and spectral patterns of five RSN classes from TFICA-SCT on the Pre-/Post-rTMS EEG data. **(A)** Visual, **(B)** auditory, **(C)** somatomotor, **(D)** frontoparietal, and **(E)** default mode networks. Group-level SCT maps were thresholded at *p* < 0.01, cluster-based correction.

Figures 2–4 illustrate spatial and spectral patterns of identified RSNs from three datasets (i.e., the EC/EO, HC/MdDS, Pre/Post-rTMS datasets), which were further categorized into five groups, i.e., visual (V), auditory (A), somatomotor (M), frontoparietal (F), and default mode (DMN) networks, based on criteria described above (see the section Evaluation and Validation Protocols). These five groups of RSNs were detected from all three datasets with high spatial and spectral similarities, while some condition-dependent variations were also observed.

In each RSN group, more than one EEG network was obtained by TFICA-SCT with significant spatial resemblance identified, each of which was termed as a subnetwork of the group. For the visual network, 4, 5, and 4 subnetworks were detected in EC/EO, HC/MdDS and Pre-/Post-rTMS, respectively (Figures 2–4). They were generally associated with the primary visual cortex (i.e., V-d in EC/EO, V-e in HC/MdDS, and V-b in Pre-/Post-rTMS), middle temporal visual area (i.e., V-a and V-b in EC/EO, V-b and V-d in HC/MdDS), V2/V3 (i.e., V-c and V-d in Pre-/Post-rTMS), and parts of the ventral stream of visual systems (other subnetworks in Figures 2–4; Goodale and Milner, 1992). Some of these subnetworks had bilateral symmetric distribution (e.g., V-d in Figure 2, V-e in Figure 3, V-c in Figure 4). Some showed the hemispheric dominance whereas the similar dominance was found on their symmetric hemisphere in other corresponding subnetworks (i.e., V-a and V-b in Figure 2, V-a and V-c in Figure 3, V-b and V-d in Figure 3). In terms of spectral patterns, the visual RSNs were characterized with an evident peak in the alpha band.

For the auditory network, 4, 3, and 3 RSNs were detected in three datasets, respectively (Figures 2–4). These RSNs mainly covered the temporal cortex, with either lateralized distribution (i.e., A-a, A-b, and A-d in EC/EO, and A-a and A-b in Pre-/Post-rTMS) or bilateral distribution (i.e., all in HC/MdDS and A-c in Pre-/Post-rTMS). Some were symmetric in their spatial patterns (e.g., A-a and A-b vs. A-c and A-d in EC/EO). Note that the bilateral symmetric subnetworks in HC/MdDS had opposite correlation values in the two hemispheres (A-a and A-b in HC/MdDS). Most RSNs had peaks in the alpha band while some showed additional peaks in the beta band.

For the somatomotor network (Figures 2–4), 2, 4, and 3 subnetworks were detected in three datasets, respectively. Two subnetworks in EC/EO covered the premotor and primary motor cortices. Four subnetworks in HC/MdDS covered different areas, i.e., M-b over the primary motor and premotor cortices, M-c over the somatosensory cortex, and M-a and M-d over the lateral primary motor cortices. The subnetworks in Pre/Post-rTMS had similar patterns compared to the subnetworks in HC/MdDS. Most of these subnetworks showed lateralized distributions (e.g., M-a and M-b in Figure 2, M-a and M-d in Figure 3, M-a and M-b in Figure 4) or lateralized dominance (e.g., M-b and M-c in Figure 3), while only one bilateral distribution was observed (i.e., M-c in Figure 4 Pre-/Post-rTMS). In terms of spectral patterns, distinct from auditory and visual networks, somatomotor subnetworks showed more peaks and higher amplitudes in the beta band than in the alpha band.

For the frontoparietal network (Figures 2–4), 6, 2, and 3 subnetworks were detected in three datasets, respectively. They mostly covered the prefrontal cortex with either unilateral (e.g., F-a and F-c in EC/EO) or bilateral (e.g., F-b in EC/EO) distributions. For the unilateral subnetworks, symmetric pairs could be found in all three datasets (e.g., F-a and F-c in Figure 2, F-a and F-b in Figure 3, F-a and F-b in Figure 4). Several unilateral subnetworks (F-a and F-b in Figure 3, F-a and F-b in Figure 4) and one bilateral subnetwork (F-b in Figure 2) showed activity on both the lateral side(s) and the medial wall(s) of the hemisphere(s). Opposite correlation values were observed in F-d in EC/EO covering the left prefrontal cortex. These subnetworks had dominant peaks in the beta band with a few in the alpha band in their spectral patterns.

For the DMN (Figures 2–4), 1, 3, and 6 subnetworks were detected in three datasets, respectively. These subnetworks exhibited more complicated spatial patterns than the other four networks. The only one detected in EC/EO covered the bilateral posterior cingulate cortex (PCC). Three subnetworks in HC/MdDS covered the medial prefrontal cortex (mPFC) and the bilateral PCC. Six subnetworks detected in Pre/Post-rTMS (Figure 4) covered the mPFC (D-a, D-b, and D-c), the inferior parietal lobe (IPL, all subnetworks except D-a), and PCC (D-d and D-e). In addition, a subnetwork (D-c) showed strong negative correlations between the mPFC and both the right PCC and the left IPL. Negative correlations were also observed in D-b between the mPFC and the right IPL. The spectral powers of all these networks showed peaks in the alpha band. Some from the Pre/Post-rTMS dataset also had another peak in the beta band.

### TFICA-SCT derived RSNs vs. fMRI derived RSN templates

**Figure 5:**
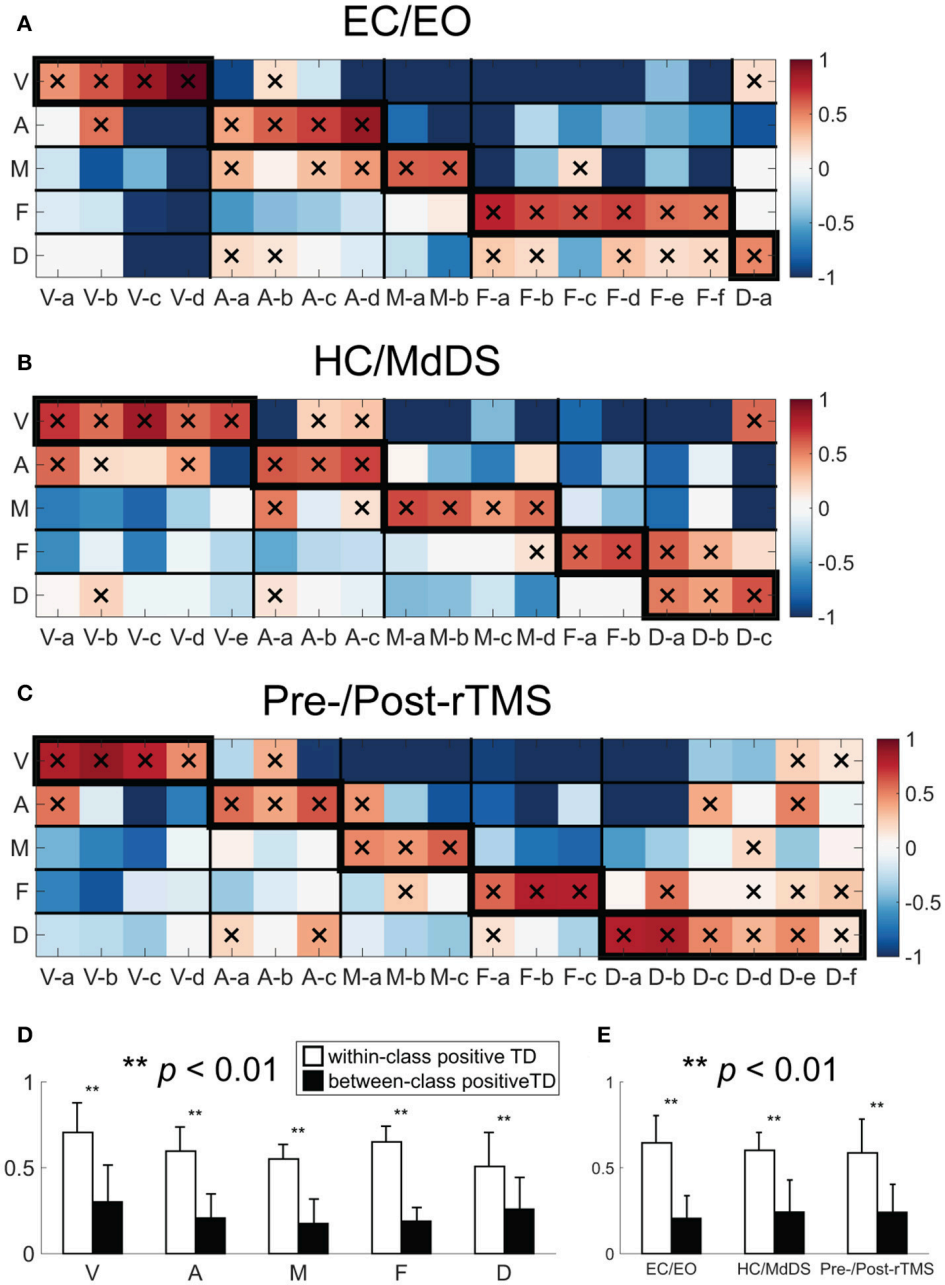
Template-matching degree (TD) between TFICA-SCT derived-RSNs and fMRI RSN templates in **(A)** EC/EO; **(B)** HC/MdDS; **(C)** Pre-/Post-rTMS. **(D)** Bar-plots of the within-class (enclosed with thicker boundaries) and between-class positive TDs over five RSN classes; **(E)** Bar-plots of the within-class and between-class positive TDs over three datasets. Note that only positive TD values were considered since negative values indicate dissimilarity.

TFICA-SCT derived RSNs were spatially compared to fMRI-derived RSN templates (Figure 5). Each EEG RSN was associated with an fMRI template RSN showing the largest TD. In general, all EEG RSNs in these five groups had significant TD values (*p* < 0.01, Bonferroni corrected) to their corresponding fMRI template RSNs (marked with “X” in the boxes along the diagonal tiles in Figures 5A–C). Similar patterns of spatial similarity was observed across three datasets. This observation was supported by the comparison of the within-class and the cross-class TD values, in which the within-class TD values were significantly higher than the cross-class TD values (Figure 5D, *t*-test, *p* < 0.01) for all five network classes. For each dataset, the within-class TD values were always significantly higher than the between-class TD values when all network classes were considered together (Figure 5E, *t*-test, *p* < 0.01). Among different groups of RSNs, the DMN and frontoparietal networks showed competing similarity to certain extent (Figures 5A–C).

### Consistency of RSNs across different datasets

**Figure 6:**
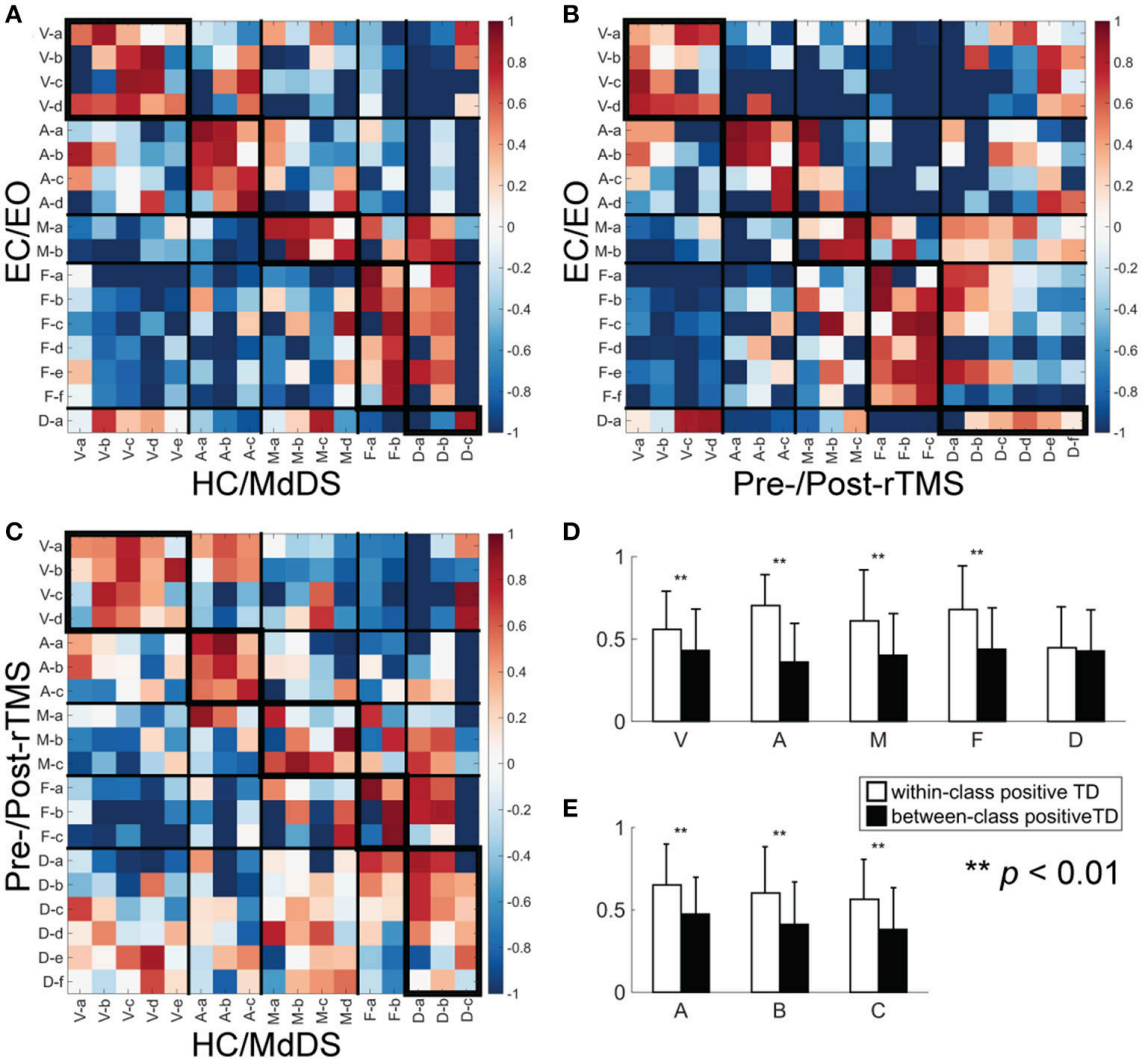
TD between a pair of SCTs from two different datasets. **(A)** EC/EO vs. HC/MdDS; **(B)** EC/EO vs. Pre-/Post-rTMS; **(C)** HC/MdDS vs. Pre-/Post-rTMS; **(D)** Bar-plots of the within-class (enclosed with thicker boundaries) and between-class positive TDs over five RSN classes; **(E)** Bar-plots of the within-class and between-class positive TDs over three datasets.

The results of pair-wise RSN comparisons from different datasets are shown in Figure 6A (for the comparison between EC/EO and HC/MdDS), Figure 6B (between EC/EO and Pre/Post-rTMS), and Figure 6C (between HC/MdDS and Pre/Post-rTMS). It is noted that significantly high spatial similarity can be observed in RSNs belonging to the same classes among three datasets. This was reflected in more positive TD values in the diagonal tiles than in the off-diagonal tiles, which were significant difference (Figure 6D, *t*-test, *p* < 0.01) for the visual, auditory, somatomotor, and frontoparietal networks when all TD values (Figures 6A–C) from three comparisons were included. For each pair-wise comparison, TD values in the diagonal tiles are higher than those in the off-diagonal tiles when all network classes were considered (Figure 6E, *t*-test, *p* < 0.01).

In terms of spectral powers, the alpha peak in the visual networks and the beta peaks in the frontoparietal networks were consistent across the three analyses. Additionally, peaks in both the alpha and the beta bands were consistently identified in both auditory and the somatomotor networks. The power spectra of the DMN revealed alpha peaks in all three analyses, though its amplitude in EC/EO was relatively low compared with other two. The DMN in Pre/Post-rTMS showed more activity in the beta band as well, which was not obvious in the other two analyses, especially in those subnetworks including the posterior cortex. These results confirmed the similarity within the same classes of RSN in both spatial and spectral patterns.

### Contrast resolutions of EEG RSNs revealing condition differences

**Figure 7:**
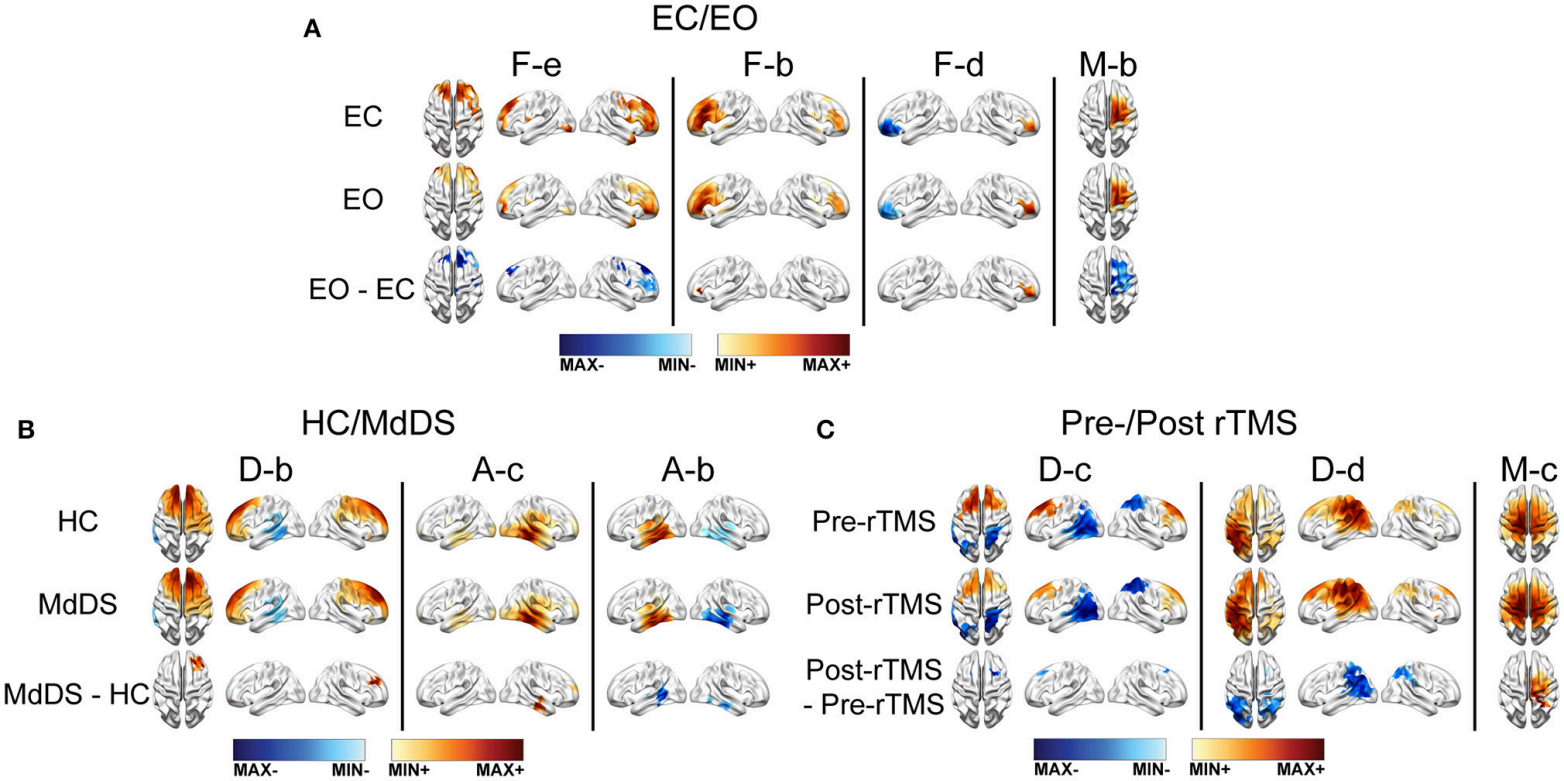
SCT differences between data from two conditions in one dataset. **(A)** EC/EO. **(B)** HC/MdDS. **(C)** Pre-/Post-rTMS. Maps were thresholded *p* < 0.01, cluster-based correction.

The spatial differences of EEG RSNs in the three datasets (i.e., EC vs. EO in the EC/EO dataset, HC vs. MdDS in the HC/MdDS dataset, and Pre-rTMS vs. Post-rTMS in the Pre/Post-rTMS dataset) are illustrated in Figure 7. Their spectral power differences are summarized in Table 1.

#### Differences of RSNs between eyes-closed and eyes-open

Four subnetworks associated with the frontoparietal (i.e., F-b, F-d, F-2) and somatomotor (i.e., M-b) RSNs were detected with significant differences (*p* < 0.01, cluster-based correction) (Figure 7A). Among them, significantly higher SCCs were detected in both left and right lateral prefrontal cortices (i.e., F-b and F-d), and significantly lower SCCs were detected in mPFC (i.e., F-e) in EO than in EC. In addition, the SCCs in the right motor areas were lower in EO than in EC. Regarding the spectral power (Table 1), significantly reduced powers in the theta and alpha bands were detected in the visual and somatomotor networks (visual theta: *p* < 0.001, visual alpha: *p* < 0.01, somatomotor theta: *p* < 0.001, motor alpha: *p* < 0.005), significantly enhanced alpha power (*p* < 0 .001) in the frontoparietal network, and significantly enhanced beta powers (*p* < 0.001) in the visual, somatomotor, frontoparietal, and DMN networks were detected in EO as compared to EC.

#### Differences of RSNs between HC and MdDS

In the comparison of HC and MdDS patients, significant differences (*p* < 0.01, cluster-based correction) (Figure 7B) were detected in two auditory subnetworks and one DMN subnetwork. MdDS patients indicated significantly higher SCCs in the right temporal cortex and significantly lower SCCs in the left temporal cortex (i.e., auditory RSNs). Significantly higher SCCs were observed over mPFC. The differences in spectral powers were not statistically compared since the paired *t*-test was not applicable to this dataset.

#### rTMS induced RSN changes

Three RSNs indicated significant rTMS induced changes between the Pre-TMS and Post-TMS conditions (Figure 7C). Specifically, following the rTMS treatment, it is observed with reduced SCCs in the bilateral mPFC nodes of a default-mode subnetwork (i.e., D-c) that showed negative connections between the frontal and parietal cortices, reduced SCCs in the bilateral IPL nodes of another DMN (i.e., D-d) that indicated positive connections between the frontal and parietal cortices, and enhanced SCCs in the right motor cortex in a somatomotor subnetwork showing bilateral patterns. Two classes of RSNs indicated significantly detected power changes, i.e., significantly enhanced theta power (*p* < 0.05) in the auditory network and significantly reduced alpha power (*p* < 0.001) in the DMN network, following rTMS treatment.

## Discussion

In the present study, a new data-driven analysis framework, termed as TFICA-SCT, was developed to directly probe RSNs from EEG data. The proposed framework combined EEG source imaging, ICA in the time-frequency domain and statistical correlation analysis, allowing the reconstruction of RSNs over a broad frequency range with unsurpassed high temporal resolution, as compared to RSN definitions from fMRI data of low resolutions in both time and frequency domains. Meanwhile, the method provided statistical power for subject-specific spatial patterns of RSNs. The proposed method was evaluated using three datasets of representative experimental conditions, i.e., eye-open vs. eyes-closed in healthy subjects, health controls vs. MdDS patients, and pre- vs. post-rTMS in MdDS patients. Various aspects of performance of the proposed framework, i.e., capability of identifying multiple RSNs, their spatial and spectral properties, consistency, and robustness, were assessed. TFICA-SCT identified five groups of major resting-state networks, i.e., visual, auditory, somatomotor, frontoparietal, and default mode networks. These RSNs were found with significant and consistent spatial similarity to fMRI RSNs. Their spatial and spectral consistencies and detection robustness were suggested from comparisons among three different datasets. Identified RSNs further revealed condition-specific changes in both spatial and spectral domains for the three compared experimental conditions.

It is noted that the proposed SCT introduces a statistical framework that includes correcting false cross-correlations from autocorrelation and cluster-based statistical thresholding in constructing the tomography of RSNs. Many of these have already been adapted in fMRI (Woolrich et al., 2001; Rombouts et al., 2005; Roy et al., 2009), but were used for the first time in creating EEG/MEG RSN tomography in our algorithm. The introduction of correlation-based statistical analysis provides two merits. First, it provides statistical quantitative metrics to be further evaluated using such a non-parametric statistical test, thereby identifying regions that significantly belong to specific RSNs. Second, it provides a means to obtain subject-specific spatial patterns, which are not readily yielded by the group-level ICA. Based on subject-specific spatial patterns, group-level inference can be made about spatial differences of RSNs because of different conditions. The effect of autocorrelation on generating false cross-correlations has been considered in the present study, as in resting-state fMRI studies (Woolrich et al., 2001; Rombouts et al., 2005; Honey et al., 2009; Roy et al., 2009). This is particularly important for EEG RSN estimations since the oscillatory nature of EEG signals suggests potentially high autocorrelations. The non-parametric statistical test is based on Monte Carlo simulations (Smith and Nichols, 2009), which utilize a cluster-based thresholding technique to address the multiple comparison problem. While the considerations behind these statistical and correlation analyses are similar to those used in 3D volumetric fMRI data, their implementation in EEG/MEG RSN estimations is new and distinct in terms of the data domain, which is a highly convoluted 2D surface, i.e., the cortex.

Among three sets of experimental data, five major resting-state networks, including visual, auditory, somatomotor, frontoparietal, and default mode networks, have been identified and represented with different numbers of subnetworks (Figures 2–4). The spatial patterns of these identified RSNs indicate high spatial similarity to RSN templates from fMRI (Figure 5; Yeo et al., 2011), as well as to fMRI RSNs reported in other literature (Smith et al., 2009, 2012; Liu and Duyn, 2013; Richiardi et al., 2015). These RSNs further indicate high spatial similarity among data from three experiments (Figure 6), while contrast-dependent variations can still be observed in both spatial (Figure 7) and spectral features (Table 1). All these results demonstrate the effectiveness and robustness of the proposed framework in identifying the cortical-level RSNs directly from EEG across different cohorts of individuals. These findings are in line with previous reports that found RSNs in EEG data (Yuan et al., 2016; Liu et al., 2017) and/or MEG data (de Pasquale et al., 2010; Brookes et al., 2011; Hipp et al., 2012). Previous EEG/MEG RSN research and advancements in TFICA-SCT indicate that RSN organizations can be retained from body surface recordings of brain signals via modeling and computation. The remarkable similarity between RSNs identified in fMRI and electrophysiological recordings (i.e., EEG/MEG) converges on the notion that RSNs as network-level organizations of distributed neural activity represent a fundamental aspect of brain physiology that are reflected in electrical and hemodynamic brain signals.

It is observed that the detection of EEG RSNs suggests several differences as compared with fMRI RSNs. Our results (Figures 2–4) revealed both lateralized RSNs and bilateral RSN from EEG, with a greater number of lateralized than bilateral RSNs, while fMRI literature indicates more symmetric and bilateral RSNs (Damoiseaux et al., 2006; De Luca et al., 2006; Agosta et al., 2012). However, this fact does not suggest inaccuracies in EEG RSNs results. First, lateralized RSNs from TFICA-SCT are consistent with resting EEG data analysis in the electrode domain, in which lateralized potential distributions toward either the left or right hemisphere have been reported (Yuan et al., 2012; Ding et al., 2014). Second, while an EEG RSN is lateralized, another symmetric EEG RSN on the contralateral hemisphere can be found (Figures 2–4). This difference might be due to the intrinsic differences in brain electrical and hemodynamic signals, in which EEG is more dynamic and directly linked to underlying network communication mechanisms than fMRI (Mantini et al., 2007; Laufs, 2008; Yuan et al., 2012). Multiple subnetworks detected for each RSN class from EEG in the present study might reflect the fact that the networking of multiple nodes (or regions) in an RSN is dynamic rather than stationary. This notion has been observed in fMRI RSNs as well (Chang and Glover, 2010; Hindriks et al., 2016), in which the architect of the whole brain network is dynamic. While most of these fMRI RSN studies reveal dynamics at the network level built on RSNs, the present study suggests that the intrinsic organization of individual RSNs are also dynamic (Fox et al., 2005; Van Den Heuvel et al., 2008; Deco et al., 2011). Furthermore, less lateralized RSN patterns in fMRI might be due to the convolution from the electrical response to the hemodynamic response, which can increase correlations among different RSNs (Yuan et al., 2016). It can be further promoted due to the global contribution of respiration and blood flow to the hemodynamic process (Wise et al., 2004; Birn et al., 2006; Shmueli et al., 2007).

The second difference between fMRI and EEG RSNs is the observation of cross-talk between EEG RSNs, especially between the frontoparietal and default mode networks. The confusion might be partially caused by the mismatch between the bilateral templates of fMRI RSNs and more lateralized EEG RSNs, as discussed above. The non-optimal selection of metrics (e.g., vectorized cortical maps for calculating spatial correlation) and protocol (e.g., unique paired match) in the analysis procedure might contribute to it as well. The cross-talk could also be partially due to the vicinity of the regions involved in these two networks. Lastly, cross-talk could be influenced by artifacts in EEG recordings and inaccuracies of modeling and computation processes in TFICA-SCT. As an example, the DMN identified from EEG is of less spatial similarity to the template compared to the other four networks. DMN constitutes multiple key regions, include mPFC, PCC, bilateral IPL (Buckner et al., 2008). Additionally, lateral temporal cortex and hippocampal cortex are often observed to be engaged as well (Damoiseaux et al., 2007). Signals from mPFC are likely to be corrupted by residual artifacts of eye movements and blinks after preprocessing (Ille et al., 2002; Joyce et al., 2004). The inverse method, i.e., MNE, has limited penetration and accuracy in estimating deep sources such as the cingulate cortex (Gorodnitsky et al., 1995; Pascual-Marqui, 1999), resulting in poor estimation of the PCC in DMN. This can be improved with more advanced ISI techniques (Liao et al., 2013; Zhu et al., 2014). The spatial smoothing effect (Baillet and Garnero, 1997; Pascual-Marqui, 1999; Babiloni et al., 2005) in the ISI process might further obscure multiple regions of DMN that are close to each other.

The high temporal resolution of EEG signals over fMRI signals enables the investigation of spectral properties of individual RSNs, which is significant in understanding human brain communication mechanisms in both healthy and sick persons (Klimesch, 1999; Rangaswamy et al., 2002; Mantini et al., 2007; Kounios et al., 2008) since invasive electrical recordings have suggested different functional roles of different brain rhythms in communication (Crone et al., 1998, 2006; Canolty et al., 2006). Results in the present study show dominant alpha band activity in visual networks, which have been observed in MEG studies (Brookes et al., 2011). The somatomotor networks have typically shown both alpha and beta spectral peaks while the frontoparietal networks have shown a strong peak in the beta band, which is consistent with MEG RSN studies (Mantini et al., 2007; Brookes et al., 2011). Strong beta activity has been reported in DMN (Laufs et al., 2003b; Mantini et al., 2007; Brookes et al., 2011), especially in mPFC (D-b and D-c in Figure 4). Furthermore, power spectra changes have been observed due to different conditions, e.g., reduced alpha power in the visual and somatomotor networks, enhanced beta power in the frontoparietal network when eyes are open (Table 1), and reduced alpha power in DMN in MdDS patients after rTMS (Table 1) (see detailed Discussions below). While data are preliminary, evidence behind them attest to the value of spectral powers of RSNs beyond spatial distributions (such as from fMRI) in understanding fundamental communication mechanisms in healthy brains and altered ones in patients. Future studies can use this additional RSN property, together with their spatial property, to investigate clinical problems in more depth.

The present results are generated based on unbiased and wide spectrum EEG data, whereas other approaches reported with MEG (Brookes et al., 2011) or combined EEG and fMRI (Goldman et al., 2002; Laufs et al., 2003a) exploit pre-determined, narrow band-passed data. The advantage of using wide-spectrum data is to give unbiased weights to activity in all frequency points, which is essential in a data-driven approach. Furthermore, many RSNs in the present study suggest activity in more than one frequency band (e.g., alpha and beta in the frontoparietal network). While the present study only includes three frequency bands (i.e., theta, alpha, and beta bands), more bands, e.g., the gamma band, which may play an important role in functional connectivity (Kounios et al., 2008; Rutter et al., 2009; Ossandón et al., 2011), can be included in future studies as necessary.

The present study further demonstrates the capability of TFICA-SCT in detecting contrast differences in conditions involving both healthy and sick persons and both at baseline and after treatment. Contrast differences are reflected in two metrics. One is the threholded SCC value after Equations (8)–(11), which indicates the affinity of a source point to a RSN (significant SCC: affinity to the RSN; non-significant SCC: no affinity to the RSN; the level of affinity indicated by the significant SCC value). The other metric is the spectral power at theta, alpha, or beta band for an RSN, which indicates the strength of an RSN that could change depending on the number of source points affined to the RSN and the accumulated strength from all affined source points. In the comparison between the eyes-closed and eyes-open conditions, the metrics of spatial affinity and spectral power indicate reduced activities in the visual and somatomotor networks with eyes open, especially in the low-frequency band (including the alpha band). The phenomenon has been well documented in literature (Raichle et al., 2001; Marx et al., 2004; Fox and Raichle, 2007; Yang et al., 2007; Jao et al., 2013). These two metrics also suggest increased beta band activities with eye open in the frontal and motor areas, which is consistent with previous EEG literature (Barry et al., 2007, 2009). Frontal areas are more active when persons are awake according to fMRI findings (Corbetta et al., 1998; Marx et al., 2004). Individuals with MdDS showed reduced spatial affinities in the left auditory RSN, which suggest hypo-connectivity that is consistent with hypo-metabolism from fMRI and PET studies, both in location and direction of abnormal connectivity (Cha et al., 2012). In the MdDS patients who responded to the treatment of rTMS, reduced spatial affinity and alpha power in the DMN RSNs were observed, lowering their hyper-connectivity in DMN (Figure 7B), which suggests the potential reason behind the responsiveness of these patients to rTMS. Reduced alpha power in DMN following rTMS, indicative of the treatment effect, has been similarly revealed in our previous study based on sensor-level EEG data (Ding et al., 2014). Enhanced theta power in the auditory RSNs (Table 1) might compensate hypo-connectivity observed in MdDS patients (A-b, Figure 7B).

Compared with fMRI RSN studies, EEG/MEG RSN studies are still at their infant stages, focusing more on technology developments in estimating spatial, temporal, and spectral patterns of RSNs (Brookes et al., 2011; Knyazev et al., 2011, 2016; Siems et al., 2016; Sockeel et al., 2016; Yuan et al., 2016; Liu et al., 2017). As discussed briefly above, studies of their functional correlates will be a significant step in the future not only for EEG, but also for MEG. The framework developed here is applicable to MEG data as well, while only EEG data have been demonstrated in the present study. EEG and MEG have their own advantages and disadvantages. MEG is less sensitive to the volume conduction effect and more sensitive to deep neural sources while EEG has better sensitive to tangential sources than MEG (Hämäläinen et al., 1993; Baillet et al., 2001). EEG can be recorded simultaneously with fMRI, which can lead to the opportunity of understanding neurophysiological underpinnings of BOLD RSNs. But, more importantly, both EEG and MEG are generated by same neuronal sources (da Silva, 2013), their integration can contribute more on accurate estimations of electrophysiological RSNs due to their complimentary nature (Ding and Yuan, 2013). Mathematically, the combination of EEG and MEG signals provide more independent measurements for the ISI step, which is expected to provide much improved mathematical solutions to associated inverse problems. Of course, both EEG and MEG face the similar challenges, such as signal leakage (Brookes et al., 2012; Hipp et al., 2012). Methods to address signal leakage have been proposed in seed-based functional connectivity estimation methods, which, however, might remove useful signals as well (Brookes et al., 2012; Hipp et al., 2012). Methods to address the same issue in ICA-based functional connectivity estimations still need to be developed. One possibility is to increase the performance of ISI (Liao et al., 2013; Zhu et al., 2014) in reducing signal leakage in inverse solutions.

The present study is limited in the following aspects. First, the categorization of SCTs into RSNs was not fully quantitative and objective. While fMRI templates were used, the quantitative metric, i.e., vectorized spatial correlation, and the protocol of unique matching were not optimal and, therefore, visual inspections were still used. Fully automated procedures need to be established in defining various classes of RSNs in the future. Second, the detection of DMN was less precise than other RSNs, which might be due to its more diffused distribution and because of the deeper spatial location of its major hubs. This could be a limitation in modeling and computation, though could be improved by several advanced modeling (Fuchs et al., 1998; Wolters et al., 2006; Friston, 2009), inverse computation (Liao et al., 2013; Zhu et al., 2014), and data-driven techniques (Cichy et al., 2016, 2017). Third, our experimental data had relatively small numbers of participants and/or only included female participants. Some of the findings in the present study might not be generalizable to males because of the gender differences in resting states (Gur et al., 1995; Kilpatrick et al., 2006; Tian et al., 2011). The proposed TFICA-SCT framework needs to be tested in data with large numbers of participants to enhance the statistical power on findings. Fourth, while the comparison between eye-open and eye-closed had suggested some information about functions of various RSNs, it is important to use more formal protocols, such as combining resting states and relevant tasks (Connolly et al., 2016) and use of patient groups with well-defined functional deficit (Tie et al., 2014), to study these networks' functional correlates. Fifth, no direct comparisons between our proposed framework and other existing works are performed in the present study, especially those from MEG studies (Brookes et al., 2011; Nugent et al., 2015; Liu et al., 2017). This is because such comparisons are very complicated since our current framework involves three major components, i.e., ISI, ICA, and correlation analysis, and each component has a group of variants in their implementations. Furthermore, the statistical correlation analysis is introduced to the current framework from fMRI, which is relatively new and has not been used in EEG/MEG RSN studies (Brookes et al., 2011; Yuan et al., 2016). Nevertheless, the comparisons with other methods will be conducted in future studies.

## Conclusion

A new framework termed as TFICA-SCT, integrating inverse source imaging, data-driven method, and statistical correlation analysis, is proposed to probe resting-state networks from scalp recordings in human brain electrical signals. The present study has evaluated the proposed framework in three experimental datasets to reconstruct, study, and compare RSNs in both healthy persons and sick individuals. Results of the present study showed that the proposed framework can reconstruct large-scale, network-level organization of spontaneous brain activity that significantly resembles the spatial patterns of fMRI RSNs. Reconstructed EEG RSNs are able to reveal condition-dependent variations in both spatial and spectral domains. Its capability in estimating RSN spectral properties is further beyond the capability of fMRI in studying RSNs and the present results suggest that these spectral properties could be used to segregate healthy individuals with those with a clinical diagnosis. Since EEG can be recorded simultaneously with fMRI, the combination of EEG and fMRI suggests a more powerful tool in understanding human brain networks, with unsurpassed spatial, temporal, and spectral resolutions. Clinical uses of these technologies are of significant potential values in identifying biomarkers for various neurological and psychiatric disorders, both for diagnosis and for treatment monitoring.

## Author contributions

CL developed the method, developed the analysis protocol, analyzed the data, wrote the manuscript. GS, SS, WB developed the analysis protocol, wrote the manuscript. HY, Y-HC designed the experiment, collected the MRI and EEG data, wrote the manuscript. LD developed the method, designed the experiment, developed the analysis protocol, wrote the manuscript.

### Conflict of interest statement

The authors declare that the research was conducted in the absence of any commercial or financial relationships that could be construed as a potential conflict of interest.
